# PKC-ζ mediated reduction of the extracellular vesicles-associated TGF-β1 overcomes radiotherapy resistance in breast cancer

**DOI:** 10.1186/s13058-023-01641-4

**Published:** 2023-04-07

**Authors:** Fayun Zhang, Zifeng Zheng, Luoyang Wang, Wenfeng Zeng, Wenjing Wei, Chunling Zhang, Ziran Zhao, Wei Liang

**Affiliations:** 1 Protein and Peptide Pharmaceutical Laboratory, Institute of Biophysics, Chinese Academy of Sciences, Beijing, 100101 China; 2 University of Chinese Academy of Sciences, Beijing, 100049 China; 3 School of Basic Medicine, Qingdao University, Qingdao, 266071 China; 4 Thoracic Surgery Department, National Cancer Center/National Clinical Research Center for Cancer/Cancer Hospital,, Chinese Academy of Medical Sciences and Peking Union Medical College, Beijing, China

**Keywords:** Breast cancer, Tumor microenvironment, Radiotherapy resistance, Regulatory T cells, Extracellular vesicles-associated TGF-β1, Protein kinase C zeta

## Abstract

**Background:**

Radiotherapy is widely applied in breast cancer treatment, while radiotherapy resistance is inevitable. TGF-β1 has been considered to be an endogenous factor for the development of radiotherapy resistance. As a large portion of TGF-β1 is secreted in an extracellular vesicles-associated form (TGF-β1_EV_), particularly in radiated tumors. Thus, the understanding of the regulation mechanisms and the immunosuppressive functions of TGF-β1_EV_ will pave a way for overcoming the radiotherapy resistance in cancer treatment.

**Methods:**

The superoxide-Zinc-PKC-ζ-TGF-β1_EV_ pathway in breast cancer cells was identified through sequence alignments of different PKC isoforms, speculation and experimental confirmation. A series of functional and molecular studies were performed by quantitative real-time PCR, western blot and flow cytometry analysis. Mice survival and tumor growth were recorded. Student’s t test or two-way ANOVA with correction was used for comparisons of groups.

**Results:**

The radiotherapy resulted in an increased expression of the intratumoral TGF-β1 and an enhanced infiltration of the Tregs in the breast cancer tissues. The intratumoral TGF-β1 was found mainly in the extracellular vesicles associated form both in the murine breast cancer model and in the human lung cancer tissues. Furthermore, radiation induced more TGF-β1_EV_ secretion and higher percentage of Tregs by promoting the expression and phosphorylation of protein kinase C zeta (PKC-ζ). Importantly, we found that naringenin rather than 1D11 significantly improved radiotherapy efficacy with less side effects. Distinct from TGF-β1 neutralizing antibody 1D11, the mechanism of naringenin was to downregulate the radiation-activated superoxide-Zinc-PKC-ζ-TGF-β1_EV_ pathway.

**Conclusions:**

The superoxide-zinc-PKC-ζ-TGF-β1_EV_ release pathway was elucidated to induce the accumulation of Tregs, resulting in radiotherapy resistance in the TME. Therefore, targeting PKC-ζ to counteract TGF-β1_EV_ function could represent a novel strategy to overcome radiotherapy resistance in the treatment of breast cancer or other cancers.

*Trial registration*: The using of patient tissues with malignant Non-Small Cell Lung Cancer (NSCLC) was approved by the ethics committees at Chinese Academy of Medical Sciences and Peking Union Medical College, Beijing, China (NCC2022C-702, from June 8th, 2022).

**Supplementary Information:**

The online version contains supplementary material available at 10.1186/s13058-023-01641-4.

## Background

Breast cancer is the leading diagnosed cancer in women and ranks the top incidence among all cancers. It is reported that up to 83% of breast cancer patients have received radiotherapy (RT) [1]. However, data over the last decade have shown that RT inevitably induces the immunosuppressive tumor microenvironment (TME), which in turn aggravates the incidences of radiotherapy resistance [2].

In the TME, transforming growth factor beta 1 (TGF-β1) is an immunosuppressive cytokine that plays an important role in the differentiation and development of Treg population [3–5]. Although the clinical data have shown that the local TGF-β1 levels in tumors would significantly increase after radiotherapy [6], it is surprising that no fluctuation of peripheral TGF-β1 has been observed [7, 8]. These findings indicate a distinct form of TGF-β1 in the breast cancer tissues that contribute to the local accumulation of TGF-β1. Traditionally, TGF-β1 is known to be secreted as latency-associated peptide-TGF-β1 (L-TGF-β1), which needs to be released from its binding proteins to form free TGF-β1 before it binds TGF-β1 receptor (TGF-βR) to activate downstream signaling pathways [9]. Recent studies, however, have revealed that, with the help of integrin ανβ8, TGF-β1 can expose the active site and binds directly to TGF-βR in its precursor form [10]. Moreover, a third form of TGF-β1 has been found as the extracellular vesicle (EV) associated-TGF-β1 (here after referred as TGF-β1_EV_) [11, 12]. In contrast to free TGF-β1 or L-TGF-β1, the functional TGF-β1_EV_ can transmit signals rapidly and effectively through endocytosis [13]. We then suppose that the elevated intratumoral TGF-β1 post-radiotherapy are associated with EVs, which cannot effectively diffuse into blood circulation due to their relatively large sizes [14].

Despite RT has been thought to promote anti-tumor immunity [15, 16], it is also reported to induce of Treg differentiation by increasing the intratumoral TGF-β1 level within the TME [17, 18]. As a major immunosuppressive regulator, the recruitment and accumulation of Tregs within TME undermines spontaneous T cell activation [19], resulting in higher risks of tumor aggressiveness, recurrence, and metastasis [20], as well as the induction of resistance to radiotherapy, and finally patients’ shorter survival [9–13]. Thus, TGF-β1 has been considered to be an endogenous factor for the development of RT resistance [21]. However, it is still unknown whether RT induced-TGF-β1 is in EV-associated form. Therefore, understanding the existing form of TGF-β1 and the immunosuppressive mechanisms of TGF-β1_EV_ in the radiated tumors could shed light on overcoming radiotherapy resistance.

Our previous work has shown that a natural flavonoid, naringenin is capable to reduce TGF-β1 secretion from breast cancer cells through inhibiting phosphorylation levels of PKCs [22]. In the present study, it is of strong necessity to explore whether naringenin exerts the same effect on reducing the secretion of TGF-β1_EV_ in the radiated tumors. As distinct PKCs have been reported to play different roles in the process of vesicles secretion in various kinds of cells [23–26], specific types of PKCs may regulate the secretion of TGF-β1_EV_ in breast cancer cells. We used a murine triple negative breast cancer model to demonstrate that irradiation promoted the release of TGF-β1 from the cancer cells, and a large proportion of the secreted TGF-β1 was in the extracellular vesicle-associated form.

Excitedly, we found that the expression of PKC-ζ was preferentially enhanced by irradiation and the blockage of PKC-ζ restricted the TGF-β1_EV_ secretion, indicating that PKC-ζ contributed to the releasing of TGF-β1_EV_. More importantly, naringenin, but not 1D11, significantly improved the radiotherapy efficacy with low side effects. The underlying mechanism of naringenin was via downregulating of the superoxide-Zinc-PKC-ζ-TGF-β1_EV_ pathway activated by radiation. Therefore, targeting PKC-ζ to counteract TGF-β1_EV_ function could represent a novel strategy to overcome radiotherapy resistance in the treatments of breast cancers or other cancers.

## Materials and methods

### Experimental animal and cell lines

Female BALB/c (6–8 weeks old) were purchased from Vital River Laboratory Animal Technology (Beijing, China). Foxp3-GFP mice were kindly provided by Prof. Yangxin Fu (University of Texas, Southwestern Medical Center, Texas, USA). All animal experiments were performed according to the institutional ethical guidelines on animal care and the protocols used for this study were approved by the Animal Care and Use Committee at the Institute of Biophysics, Chinese Academy of Sciences. Murine breast cancer 4T1 cell line was obtained from ATCC and cultured in 5% CO_2_ and maintained in RPMI 1640 medium supplemented with 10% FBS (VivaCell, Isreal) 100 U/ml penicillin, and 100 mg/ml streptomycin.

### Exocellular vesicles purification

For in vitro experiment, 4T1 cells were cultured in RPMI 1640 media supplemented with 10% EV-depleted FBS. Supernatant was collected and centrifuged at 500 g for 10 min followed by a step of 3000 g for 20 min at 4 °C to pellet cells and debris. The supernatant was collected without disturbing the cell/debris pellet and was transfered to an ultracentrifuge tube. Then the supernatant was centrifuged at 100,000 g for 70 min at 10 °C and the EV pellets were collected. The pellets were resuspended in a small volume of PBS. For tumor tissues, the harvested tumors were dissected and cut into small pieces, followed by culture in RPMI 1640 media supplemented with 10% EV-depleted FBS for 48 h. Supernatant of tumor pieces was collected for the EVs purification. The EVs was characterized by Transmission Electron Macroscopy (FEI, Tecnai Spirit, 120 kV, USA) and quantified by BCA protein assay kit.

### TGF-β1 detection by ELISA

TGF-β1 in supernatant or EV was detected by ELISA (DY1679, R&D Systems, Minneapolis, MN). In brief, the 96-well microplate was coated with the Capture antibody overnight at 4 degree. Cells were washed by filling each well with Washing buffer. The plate was blocked by Block buffer for 1 h. TGF-β1 was activated by HCl and added to each well and incubate 2 h at room temperature. Cells were washed and the detection antibodies were added and incubated for 2 h. Streptavidin-HRP was added to each well. Washing wells and adding Substrate solution to each well were followed by adding Stop solution. The optical density of each well was determined immediately at 450 nm.

### Data sources and processing

The gene mRNA expression matrix and clinical follow-up information of Breast Invasive Carcinoma patients with information of radiation therapy were obtained from the cBioPortal database (http://www.cbioportal.org). The association between TGF-β1 expression and the overall survival was determined using the Kaplan–Meier survival analysis with the 'survival' package (version 4.1.2) in R statistics software, and the Log-rank test was used to detect significant differences. Immune cell infiltration was obtained from the ImmuCellAI database (http://bioinfo.life.hust.edu.cn/ImmuCellAI#!/) and the clinical information was also acquired from the cBioPortal database by using the patient ID.

### Western blot

Cells were lysed by RIPA lysis buffer and the protein concentration was determined by BCA protein assay. Cell lysates were separated by SDS-PAGE gels and transferred to nitrocellulose membranes. The membranes were then blotted with the indicated antibodies (anti-TGF-β1 antibody, ab179695; anti-PKCζ antibody, ab108970; anti-p-PKCζ antibody, ab76129; anti-β-actin antibody, ab8226. Abcam, Cambridge, UK).

### PMA and CAL treatment

Phorbol myristate acetate (PMA) and Calphostin C (CAL) were purchased from Merck and used at the indicated concentrations. 4T1 cells were treated by PMA or CAL for 48 h. Supernatant was collected and EV was purified.

### guideRNA sequence

**Table:** 

gRNA1-F	5′–caccGTCCTACAAATAGGACGTGC-3′
gRNA1-R	5′–aaacGCACGTCCTATTTGTAGGAC–3′
gRNA2-F	5′–caccgCGACCCACGTAGTAGACGAT–3′
gRNA2-R	5′–aaacATCGTCTACTACGTGGGTCGc–3′

### TGF-β1 knockout of 4T1 cell line generation

gRNA targeting sequence were designed as the sequences described above using CRISPR design tool (https://zlab.bio/guide-design-resources) and the sgRNA oligos with BbsI restriction site were prepared by annealing. The gRNA oligos were constructed into pX458M and EZ-Guide plasmids, respectively. Digest pX458M-gRNA1 and EZ-Guide-gRNA2 plasmid using XhoI and HindIII Restriction Enzyme (New England Biolabs, Beijing, China) and the two plasmids were ligated by T4 DNA ligase. pX458M-gRNA1 + gRNA2 plasmid were transformed into 4T1 cells by Lipofectamine 3000 Reagent (Thermo Fisher Scientific, Waltham, MA). After 48 h transfection, GFP + cells were sorting into 96-well plate by FACS Influx (BD, Franklin Lake, NJ) and cells were identified by qPCR for TGFB mRNA and ELISA for TGF-β1 protein.

### Quantitative real-time PCR (qPCR) assay

Total RNA was isolated from cells by Trizol (Invitrogen, Carlsbad, CA). The mRNA was reversely transcribed to cDNA by M-MLV reverse transcriptase (Invitrogen, Carlsbad, CA). qPCR was performed using SYBR Green qPCR SuperMix (Invitrogen, Carlsbad, CA) to detect the expression of TGF-β1 and PKCs mRNA. Gene expression was normalized to GAPDH expression and presented as fold-change compared to the Control experiment.

### Primer sequence

**Table:** 

TGF-β1-F	TCGACATGGATCAGTTTATGCG
TGF-β1-R	CCCTGGTACTGTTGTAGATGGA
Prkca-F	GTTTACCCGGCCAACGACT
Prkca-R	GGGCGATGAATTTGTGGTCTT
Prkcb-F	GTGTCAAGTCTGCTGCTTTGT
Prkcb-R	GTAGGACTGGAGTACGTGTGG
Prkcc-F	CTCGTTTCTTCAAGCAGCCAA
Prkcc-R	GTGAACCACAAAGCTACAGACT
Prkcd-F	CCTCCTGTACGAAATGCTCATC
Prkcd-R	GTTTCCTGTTACTCCCAGCCT
Prkce-F	GGGGTGTCATAGGAAAACAGG
Prkce-R	GACGCTGAACCGTTGGGAG
Prkcq-F	TATCCAACTTTGACTGTGGGACC
Prkcq-R	CCCTTCCCTTGTTAATGTGGG
Prkci-F	CTTTGCAGTGAGGTTCGAGAT
Prkci-R	AGCCTCTTCTAACTCCAACTGAG
Prkch-F	TCCGGCACGATGAAGTTCAAT
Prkch-R	TACGCTCACCGTCAGGTAGG
GAPDH-F	AGGTCGGTGTGAACGGATTTG
GAPDH-R	TGTAGACCATGTAGTTGAGGTCA

### Coculture of EVs and naïve splenocytes

Anti-CD3 antibody was coated into 96-well U plate overnight. Naïve splenocytes were added into each well. EVs were isolated from cells treated by different reagents and quantified by BCA protein assay. Different EVs were diluted by same times and added to naïve splenocytes with anti-CD28 antibody. After 72 h, cells were collected, stained with fluorescent antibodies and detected by Flow cytometry.

### Immunohistochemistry

Immunohistochemical studies were done in 5-µm sections of paraffin-embedded tumor tissues using antibodies for TGF-β1 to determine its content in the tumors. The slides were incubated in citrate buffer for 20 min in a steamer and endogenous peroxidase was blocked by incubation with 3% H_2_O_2_ for 20 min at room temperature. The anti-TGFβ1 antibody (ab179695, Abcam, Cambridge, UK) was used in a dilution of 1:500. The slides were then stained with the secondary antibody in a dilution of 1:500. To determine the protein expression, stained slides were examined under fluorescence microscopy.

### PKC-ζ siRNA

4T1 cells at 80% confluence were transfected with siRNA (sc-36254, Santa Cruz) by Lipofectamine 3000 Reagent (Thermo Fisher Scientific, Waltham, MA) for 24 h. Cells were treated by PMA or radiation for indicated time.

### Treg differentiation assay

Spleen of GFP-Foxp3 transgenic BALB/c mice was harvested and single cell suspension was prepared. Cells were treated with anti-CD3ε/CD28 function antibody and EV or TGF-beta1 or 1D11 (BE0057, InVivoMab, West Lebanon, NH) for 72 h. Percentage of CD4^+^GFP^+^ cells in CD4^+^ cells was detected by BD FACSCalibur.

### Structure analysis

The following putative structures of mouse PKCs were downloaded from the AlphaFold Protein Structure Database (https://alphafold.ebi.ac.uk/ and used in our study: KPCA (UniProtKB: P20444), KPCB (UniProtKB: P68404), KPCD (UniProtKB: P28867), KPCE (UniProtKB: P16054), KPCG (UniProtKB: P63318), KPCI (UniProtKB: Q62074), KPCL (UniProtKB: P23298), KPCT (UniProtKB: Q02111), KPCZ (UniProtKB: Q02956). Geometrical alignments, as well as visualization, were performed with PyMOL version 2.1.0.

### Zinc-specific fluorescence staining

Before radiation administration, 4T1 cells were treated with Naringenin of 200 uM concentration for 30 min. X-Ray of 2, 4 or 8 Gy dose was used to treat 4T1 cells, respectively. After 2 h, cells were collected and washed with PBS 3 times. TSQ was dissolved in Lock’s buffer (pH 7.4) and cells were stained with 150 nM TSQ for 1 min. After 3 times washing, cells were added into 96-well plate and were examined under a fluorescence microplate reader (Excitation, 345 nm; Emission 495 nm) (SpectraMax M4 Multi-Mode Microplate Reader, Molecular Devices, San Jose, CA). The instrument measures the intensity of the reradiated light and expresses the result in Relative Fluorescence Units (RFU) using SoftMax Pro Software (Standard Edition 7.1).

### Animal model

4T1 cells were subcutaneously injected at 5 × 10^4^ cells per mouse. Mice were randomized to treatment groups. Radiotherapy was administrated with a dose of 10 Gy when tumor volume reached 70–80 mm^3^. 100 mg/kg naringenin was administrated daily by intragastric administration for 30 days. 5 mg/kg 1D11 was injected intraperitoneally 3 times per week for 3 weeks. Tumor volumes were measured twice a week and calculated as length*width*height/2. The survival days of tumor-bearing mice were recorded. All animal experiments were performed according to the institutional ethical guide lines on animal care and the protocols used for this study were approved by the Animal Care and Use Committee at the Institute of Biophysics, Chinese Academy of Sciences.

### Flow cytometry and antibodies

Single-cell suspensions were prepared. Samples were stained (20–30 min) with the following antibodies: anti-CD45, anti-CD3, anti-CD4, anti-CD8α, anti-CD25 antibodies. For intracellular staining, cells were fixed, permeabilized overnight at 4 °C (Fixation/Permeabilization Concentrate and Diluent kit, eBioscience, San Diego. CA) and subsequently stained using anti-Foxp3 antibody for 30 min. All experiments were performed on BD FACSCalibur or BD LSRFortessa and data was analyzed with FlowJo 7.6.1.

### Statistical analysis

Student’s t test was used for comparisons of datasets with two groups. For multiple comparisons, we used type II ANOVA with correction of statistical hypothesis testing. Statistical significance was considered reached for *p* values < 0.05. Survival was analyzed by Log-rank (Mantel-Cox) test.

## Results

### TGF-β1 neutralizing antibody 1D11 only partially improved the radiotherapy effect on 4T1 breast cancer

**Fig. 1:**
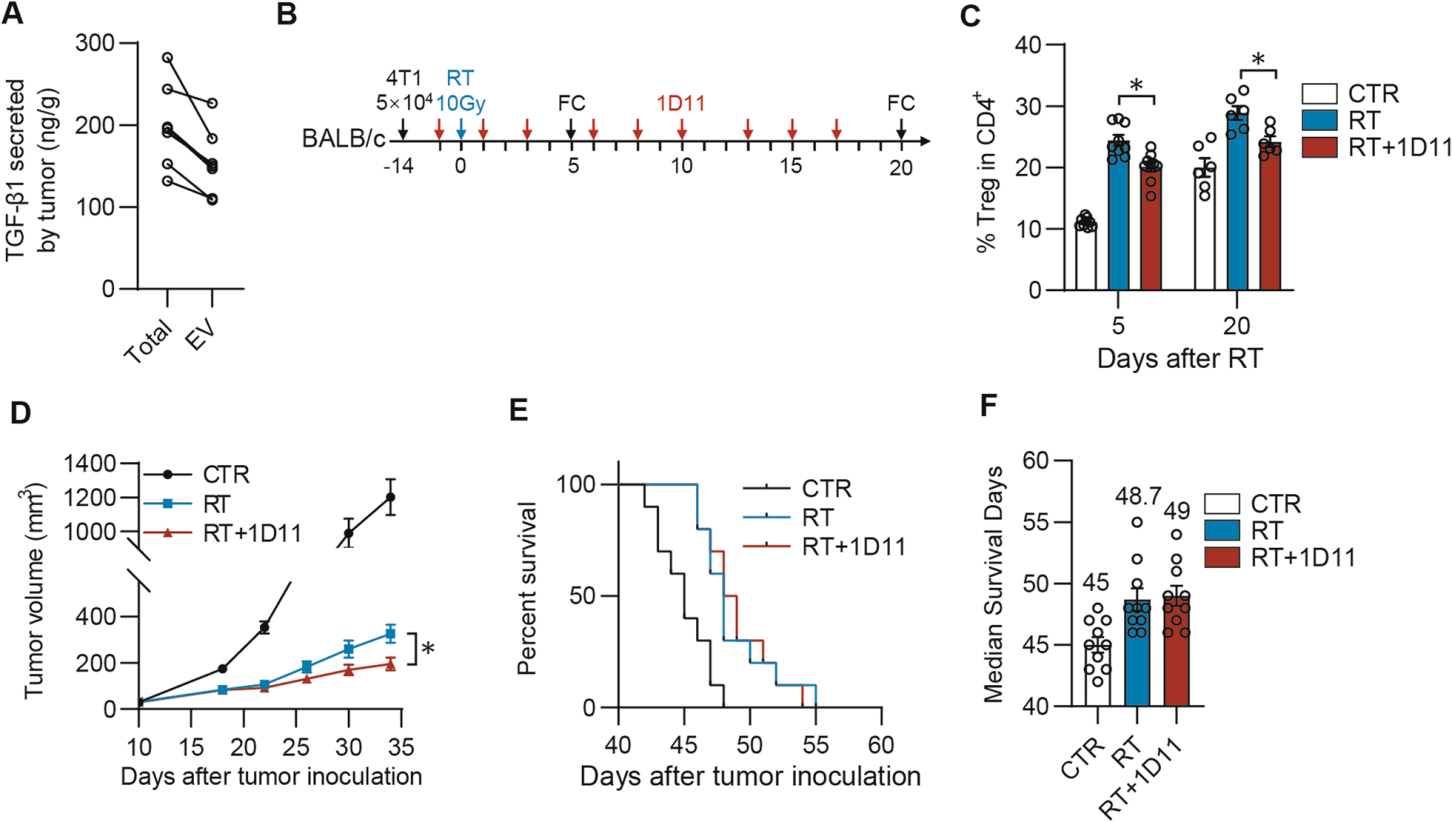
1D11 improves radiotherapy effects on the tumor growth but not life span in murine 4T1 breast cancer model. **A** TGF-β1 concentration in EVs secreted by NSCLC tumor specimen. Tumor tissues from NSCLC patients were cultured in vitro for 48 h. Culture medium was collected for EV separation and ELISA was performed for analysis of TGF-β1 *n* = 6. **B** Schematic diagram of tumor therapy model. Tumor model was established by injecting 5 × 10^4^ 4T1 cells subcutaneously into the fourth mammary pad of BALB/c mice. Radiation (10 Gy) was performed 14 days after injection of tumor cells. Mice were treated by anti-TGF-β1 antibody (1D11, 10 mg/kg) intravenously 3 times per week for 3 weeks. **C** Tumor tissues were collected at indicated day for flow cytometry analysis. Percentage of CD25^+^Foxp3^+^ (Treg) in CD4.^+^cells *n* = 6. **D** Tumor volume was recorded *n* = 15. **E** Survival curve in groups of different treatment combination *n* = 10. **F** Median survival time in groups of different treatment combination *n* = 10. Experiments was analyzed by 2-way ANOVA. *p < 0.05

As RT has been reported to cause the increases of the intratumoral TGF-β1 level and the infiltration of Tregs in the TME [17, 18], TGF-β1 therefore is considered to be an endogenous factor for RT resistance. To investigate how much TGF-β1 is secreted in the form associating with EVs in clinical settings, the EVs of tumor samples were isolated from six NSCLC patients, respectively. As expected, up to 72% of TGF-β1 was secreted in the EV-associated form in these tumors from the NSCLC patients (Fig. 1A).

Given the clinical data indicate that the TGF-β1 level is increased in the irradiated tumors [8, 27], we then wondered if the increased TGF-β1 could augment Tregs infiltration in tumors after irradiation. In line with the previous finding, the irradiation was found to promote more Tregs infiltration in 4T1 tumors than the untreated control (Fig. 1B, C). Curiously, compared to RT alone, the combination of TGF-β1 neutralizing antibody 1D11 only partially suppressed radiation-induced Tregs infiltration (Fig. 1C). A delayed tumor growth was observed after RT and 1D11 combination treatment (Fig. 1D), while the survival benefit was comparable to the mice received RT alone (Fig. 1E, F). These results suggested that 1D11 did not effectively block the function of the TGF-β1_EV_ to improve the effect of radiotherapy.

### TGF-β1 in tumor-derived EVs induce Treg differentiation

**Fig. 2:**
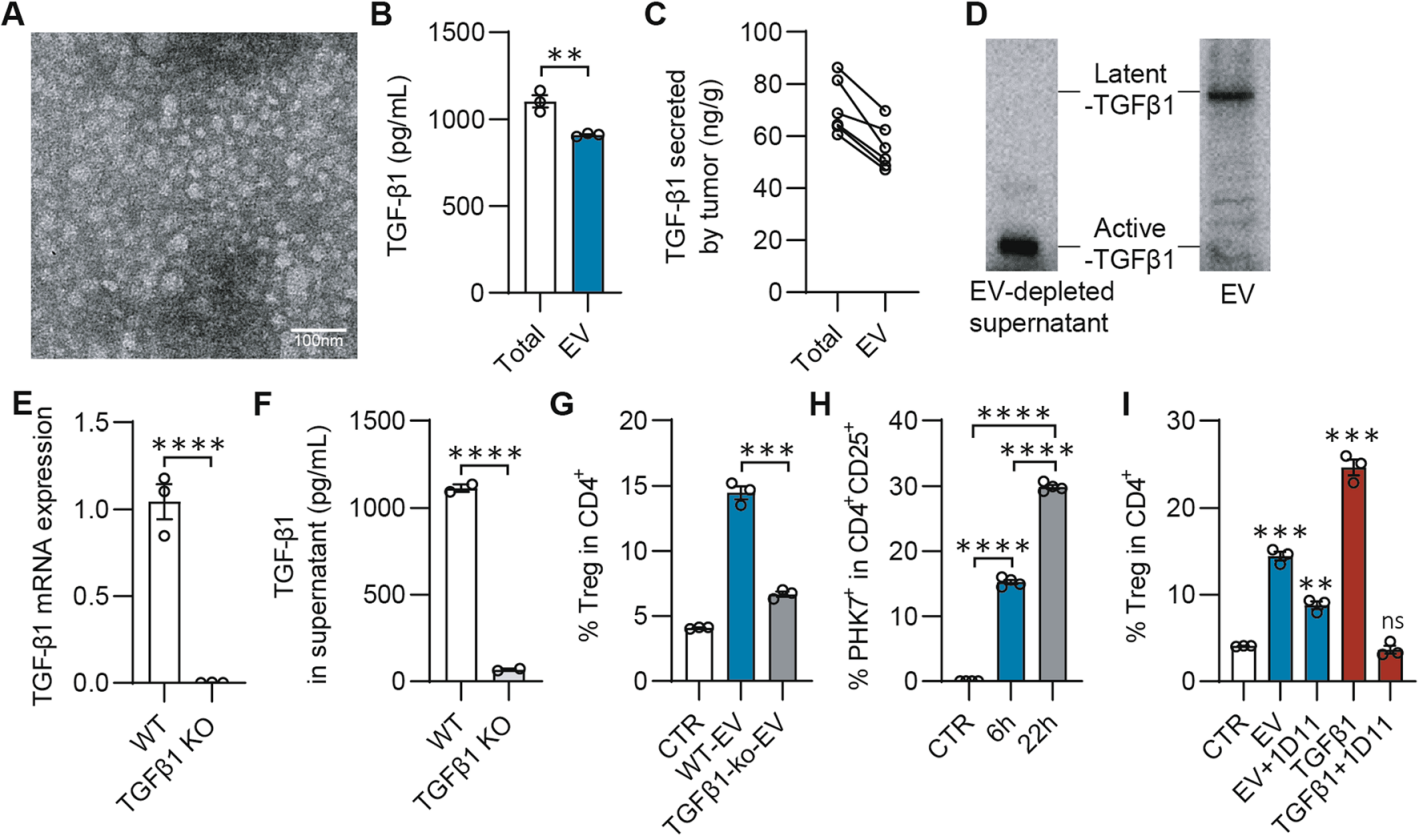
Tumor-derived EVs induce differentiation of T cells into Treg through TGF-β1 in Vitro. **A** Transmission electron microscopy (TEM) image of EVs secreted by 4T1 cells. Scale bar, 50 nm. **B** TGF-β1 concentration in total supernatant or EVs of 4T1 cells. **C** TGF-β1 concentration in total supernatant or EVs secreted by tumor pieces. 4T1 tumor model was established by injecting tumor cells subcutaneously into the fourth mammary pad of BALB/c mice. After 35 days, tumor tissues were collected, cut into pieces and cultured in vitro for 48 h. Culture medium was collected for EV isolation and ELISA was performed for analysis of TGF-β1. **D** Western blot analysis of TGF-β1 in EV or EV-depleted supernatant from 4T1 cells. **E** qPCR analysis of TGF-β1 mRNA in WT or TGFβ-1-ko 4T1 cell line. **F** ELISA analysis of TGF-β1 in supernatant from WT or TGFβ1-ko 4T1 cell line. **G**, **H** Naïve lymphocytes from the spleen of Foxp3-GFP transgenic mice were treated by anti-CD3/CD28 functional antibody. **G** The EVs secreted by WT or TGF-β1-ko 4T1 cells were added to the lymphocytes for 72 h and percentage of CD4^+^GFP^+^ cells (Tregs) were analyzed by flow cytometry. **H** Percentage of PHK^+^ cells in CD4^+^CD25^+^cells. EVs secreted by 4T1 cells were isolated and labeled with PHK. Naïve spleen cells from C57BL/6 mice were stimulated by anti-CD3/CD28 functional antibody and incubated with 500 µg/ml of PHK-labeled EVs for 72 h and percentage of PHK^+^ cells in CD4^+^CD25^+^cells were analyzed by flow cytometry. **I** The EVs secreted by WT or TGF-β1(5 ng/mL) with or without 1D11 (10 ng/mL) were added to the lymphocytes for 72 h and percentage of CD4^+^GFP^+^ cells (Tregs) were analyzed by flow cytometry. *p < 0.05; **p < 0.01; ***p < 0.001; ****p < 0.0001. **B**, **E**, **F** were analyzed by T-test. **G**–**I** were analyzed by 2-way ANOVA

In order to verify the functions of the TGF-β1_EV_ secreted by breast cancer cells, we isolated EVs from 4T1 cells and characterized. The results showed that the average diameter of the EVs was about 40 nm (Fig. 2A), and approximately 82% of TGF-β1 secreted by 4T1 cells was associated with the EVs (Fig. 2B). Consistent with the finding in 4T1 cells, up to 80% of the TGF-β1 was secreted with EVs from 4T1 tumor tissues of balb/c mice (Fig. 2C). Notably, we found that the TGF-β1_EV_ was mainly in the form of latent-TGF-β1, while the TGF-β1 left in the EV-free supernatant derived from 4T1 cells was mainly in the form of active-TGF-β1 (Fig. 2D). The latent TGF-β1_EV_ transmits signals after endocytosis [13, 28], which probably explained why 1D11 or other TGF-β1 inhibitors to bind ligands or receptors could not effectively block the function of the TGF-β1_EV_.

To further determine if the TGF-β1_EV_ affect T cells differentiation, we generated the TGF-β1 knockout (KO) 4T1 cell line (Fig. 2E, F). A significant increase of Tregs population in response to the EVs from wild type (WT) 4T1 cells were observed. The TGF-β1 knockout impeded the ability of the EVs to induce Tregs population (Fig. 2G). However, 1D11 only had a partial effect on the TGF-β1_EV_-induced Tregs infiltration (Fig. 2I). We further demonstrated that the EVs from 4T1 cells directly promoted the differentiation of splenic CD4^+^ T cells into Tregs (Additional file 1: Fig. S1A). In addition, the TGF-β1_EV_ did not show any effects on the proliferation and apoptosis of Treg cells (Additional file 1: Fig. S1B, C). Interestingly, the PHK67-labeled TGF-β1_EV_ was rapidly engulfed by CD4^+^CD25^+^ lymphocytes and resulted in the intracellular accumulation (Fig. 2H). These data suggested that the tumor-derived EVs containing TGF-β1 is capable of inducing CD4^+^ T cells differentiation into Tregs, which cannot be effectively blocked by the TGF-β1 neutralizing antibody.

### Increased TGF-β1_EV_ correlates with Tregs accumulation in the TME

**Fig. 3:**
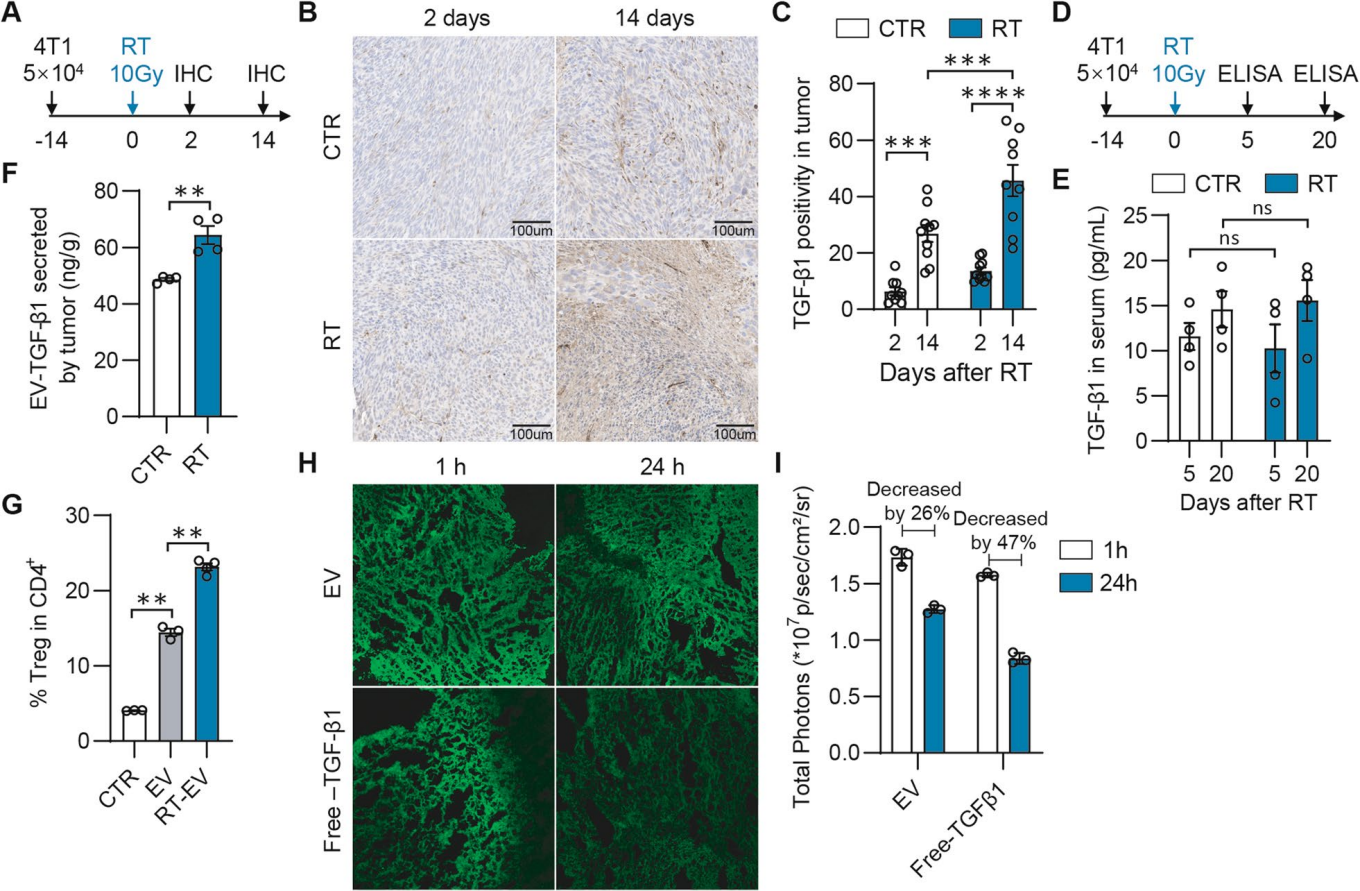
Increasing Tregs in tumor are related to increasing TGF-β1_EV_ in TME. **A** Schematic diagram of tumor model. Tumor model was established by injecting 5 × 10^4^ 4T1 cells subcutaneously into the fourth mammary pad of BALB/c mice. Tumor-bearing mice were treated with 10 Gy radiation 14 days after injection of tumor cells. Tumor tissue were collected and fixed for IHC 2 or 14 days after radiation. **B** IHC image of TGF-β1 in tumor according to **A**. Scale bar, 100 µm. **C** TGF-β1 positivity analysis in **B**. For each group, *n* = 9. Analysis was performed by Aperio ImageScope, version 12.4. **D** Schematic diagram of tumor model. Tumor model was established by injecting 5 × 10^4^ 4T1 cells subcutaneously into the fourth mammary pad of BALB/c mice. Radiation (10 Gy) was performed 14 days after injection of tumor cells. Peripheral blood was collected and TGF-β1 was analyzed by ELISA. **E** TGF-β1 protein concentration in peripheral blood in **D**. **F** TGF-β1 protein concentration in EVs secreted by tumor pieces with or without radiotherapy. Tumor-bearing mice were treated with 10 Gy radiation. 20 days after radiotherapy, Tumor tissues were collected and cultured in vitro for 48 h. Culture medium was collected for EV isolation and ELISA was performed for analysis of TGF-β1. **G** Percentage of Treg in CD4^+^cells. Naïve spleen cells from Foxp3-GFP mice were treated by anti-CD3/CD28 functional antibody and EVs secreted by 4T1 cells with or without pretreatment of radiation for 72 h and percentage of CD4^+^CD25^+^ cells were analyzed by flow cytometry. **H** Confocal imaging of tumor slides injected with fluorescence labeled EVs or free TGF-β1. 500 µg of PHK fluorescence labeled EVs or FITC-labeled free TGF-β1 were intratumorally injected. After 1 h or 24 h, frozen sections were operated and detected using confocal scanned imaging. **I** Histogram of fluorescence photons in confocal imaging slides of (H). *p < 0.05; **p < 0.01; ***p < 0.001; #*p* < 0.0001. **C**–**F** were analyzed by *t*-test. **G** was analyzed by 2-way ANOVA

To test if the TGF-β1_EV_ contributes to Tregs induced radiotherapy resistance in the TME, we employed a 4T1 breast cancer model treated by irradiation (Fig. 3A). In line with the previous reports [18], the area of TGF-β1 positive region in IHC slides was increased from 6.4% (on day 16) to 26.8% (on day 28) after tumor cell injection (Fig. 3B, C). Notably, irradiation further enlarged the area of TGF-β1 positive region from 25% (on day 2) to 45% (on day 14) after RT (Fig. 3B, C). However, the TGF-β1 concentration in peripheral blood was not changed regardless of whether the mice were irradiated (Fig. 3D, E). The irradiation-induced TGF-β1 in tumor was supposed to be also mainly in a form associated with EVs. Indeed, more than 65% of the TGF-β1 was on the EVs in both 4T1 cells and tumor tissues after irradiation (Additional file 1: Fig. S2A, B). Furthermore, compared with the control group, the irradiation promoted 4T1 tumors to release more TGF-β1_EV_ (Fig. 3F), thus resulting in more Tregs infiltration (Fig. 3G). To verify if the TGF-β1_EV_ tends to retain in TME, we intratumorally injected the fluorescent-labeled TGF-β1_EV_ and the free TGF-β1 into 4T1 tumor, respectively. The results demonstrated that the free TGF-β1 diffused away from tumor tissue much faster than the TGF-β1_EV_ (Fig. 3H). Compared with 1 h post-injection, only 53% of free TGF-β1 remained after 24 h of injection, meanwhile, much higher fluorescent intensity (~ 74%) was retained in tumors after the TGF-β1_EV_ injection (Fig. 3I). These results suggested that the retained TGF-β1_EV_ in TME highly possibly induced immunosuppression through promoting the differentiation of CD4^+^ T cells to Tregs.

### PKC-ζ masters TGF-β1_EV_ secretion upon radiation in 4T1 cells

**Fig. 4:**
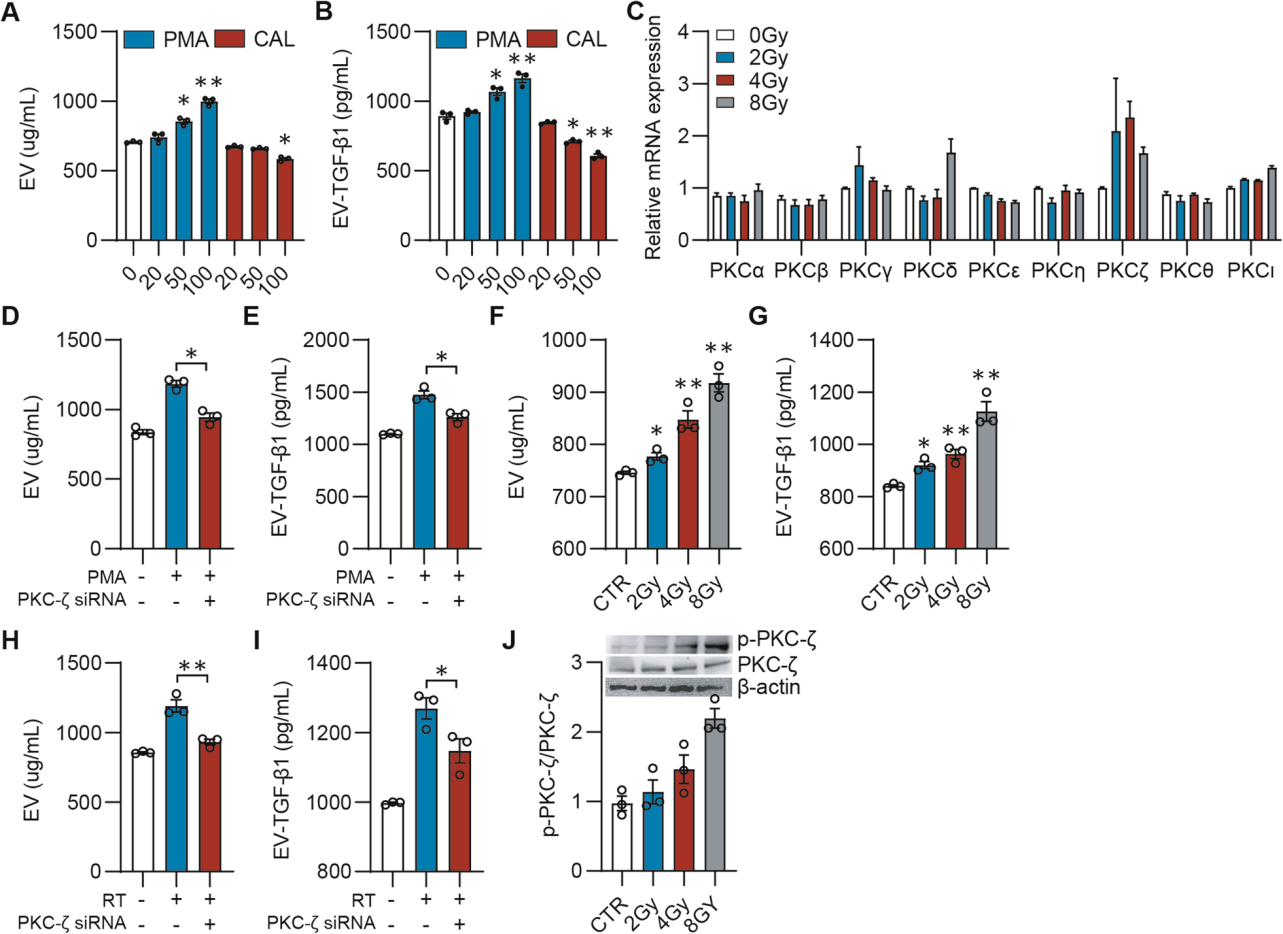
PKC-ζ regulated TGF-β1_EV_ secretion in 4T1 cells. **A**, **B** 4T1 cells were treated with 20, 50, 100 ng/mL PMA or CAL for 48 h. EVs and EV-associated TGF-β1 were quantified by BCA (**A**) and ELISA (**B**), respectively. **C** Relative mRNA expression level in responding to different dose of X-Ray. **D**, **E** 4T1 cells were transfected with PKC-ζ siRNA for 24 h followed by treatment with PMA (100 ng/mL) for 48 h. EVs and EV-associated TGF-β1 were quantified by BCA (**D**) and ELISA (**E**), respectively. **F**, **G** 4T1 cells were treated by 2–8 Gy X-Ray. EVs and EV-associated TGF-β1 were quantified by BCA (F) and ELISA (G), respectively. **H**, **I** 4T1 cells were transfected with PKC-ζ siRNA for 24 h followed by treatment with 8 Gy X-Ray. EVs and EV-associated TGF-β1 were quantified by BCA (**H**) and ELISA (**I**), respectively. **J** 4T1 cells were treated by 2–8 Gy X-Ray. Cells were collected and cell lysate were detected by western blot for PKC-ζ and p-PKC-ζ. *p < 0.05; **p < 0.01. All experiments was analyzed by 2-way ANOVA

As PKCs have been reported to be involved in the secretion of EVs, to understand the underlying mechanism of the TGF-β1_EV_ secretion, we focused on PKCs. As expected, the pan-PKCs agonist PMA promoted the TGF-β1_EV_ secretion from 4T1 cells. Conversely, the inhibition of PKCs by Calphostin C (CAL) showed an opposite effect (Fig. 4 A, B).

Radiation has been shown to promote the TGF-β1_EV_ secretion from tumor tissues (Fig. 3F). To identify the subtype of PKCs regulating the TGF-β1_EV_ secretion, we compared the mRNA expression of various PKC subtypes before and after irradiation. We found that, even after receiving a low dose of radiation as 2 Gy, the mRNA expression of PKC-ζ was elevated more significantly than that of other PKC subtypes (Fig. 4C), suggesting that PKC-ζ could effectively regulates the TGF-β1_EV_ secretion. We then used the siRNA of PKC-ζ, which was verified for its effectiveness (Additional file 1: Fig. S3), to treat 4T1 cells before treatment of PMA or radiation. The results showed that the suppression of PKC-ζ by siRNA significantly inhibited the TGF-β1_EV_ secretion induced by PMA (Fig. 4D, E). As expected, the secretions of EVs and TGF-β1_EV_ from 4T1 cells (Fig. 4F, G) and the phosphorylation level of PKC-ζ in 4T1 cells (Fig. 4J) were dose-dependently enhanced after irradiation. Accordingly, the PKC-ζ siRNA significantly reduced the TGF-β1_EV_ release induced by irradiation (Fig. 4H, I). These results indicated that PKC-ζ could play an essential role in the TGF-β1_EV_ secretion.

### Naringenin reduces TGF-β1_EV_ secretion by inhibition of PKC-ζ on phosphorylation level

**Fig. 5:**
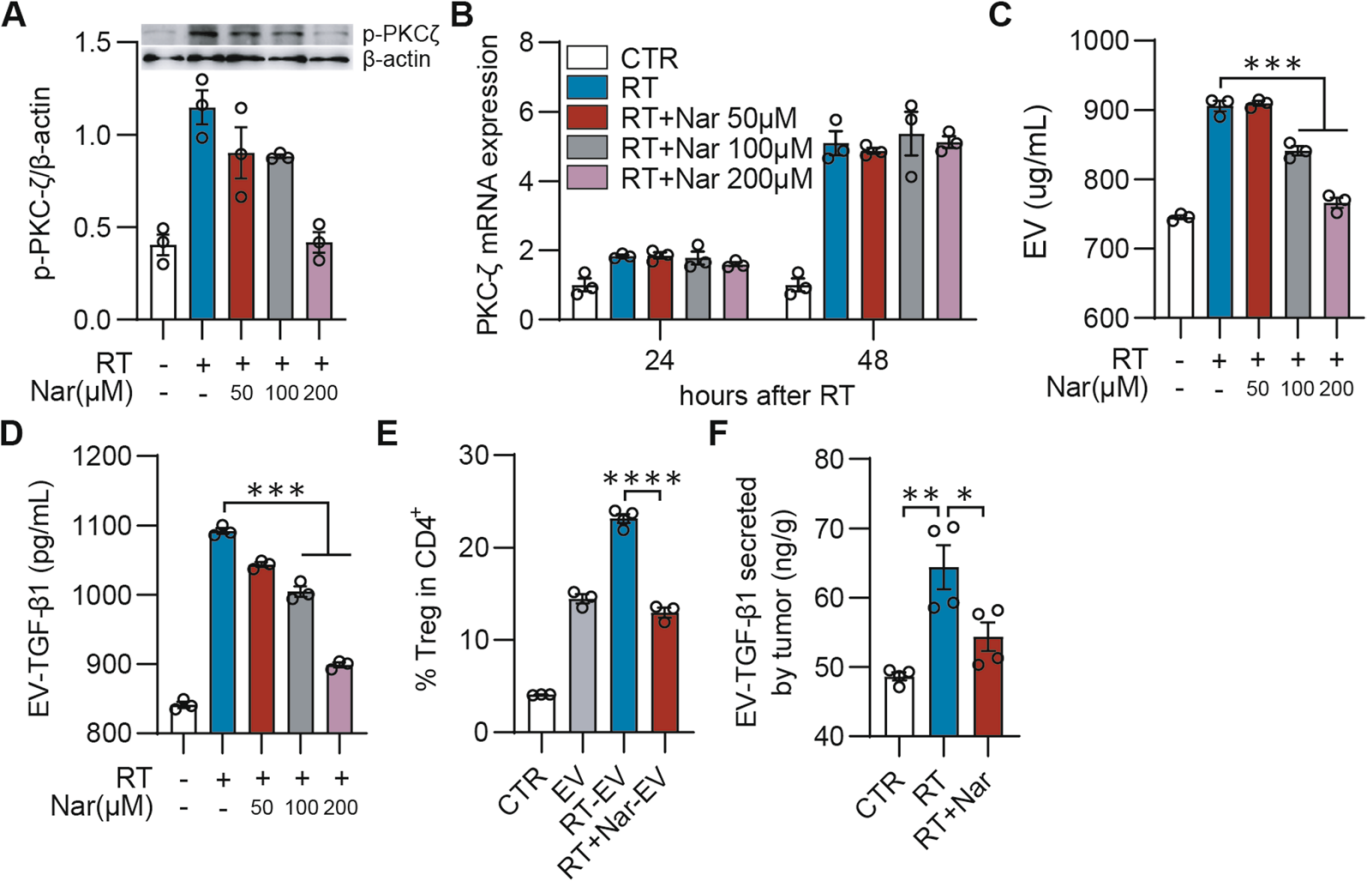
Naringenin inhibited PKC-ζ to suppress TGF-β1_EV_ secretion. **A** The phosphorylation level of PKC-ζ in 4T1 cells after treatment. 4T1 cells were treated by naringenin (Nar) in indicated concentration for 6 h followed by 8 Gy of X-Ray. The p-PKC-ζ level in cell lysate was detected by western blot. **B** The mRNA expression level of PKC-ζ. 4T1 cells were treated by naringenin in indicated concentration for 6 h followed by 8 Gy X-Ray. Total RNA was extracted and PKC-ζ mRNA expression was detected by qPCR 24 or 48 h after radiation. **C, D** 4T1 cells were treated same as **A**, **B**. EVs and EV-associated TGF-β1 were quantified by BCA (**C**) and ELISA (**D**), respectively. **E** Treg induced by EVs. 4T1 cells were treated by radiation with or without naringenin (200 µM) for 48 h and EVs in the culture medium were isolated and diluted with PBS in same volume. Naïve spleen cells from Foxp3-GFP mice were treated by anti-CD3/CD28 functional antibody with an equal volume of EVs for 72 h and CD4^+^GFP^+^ cells were analyzed by flow cytometry. **F** EV-associated TGF-β1 secreted by tumor. Tumor-bearing mice were treated by radiotherapy (10 Gy) with or without naringenin (100 mg/kg). Tumor tissues were dissected and cultured in vitro for 48 h. EVs were isolated and TGF-β1 in EVs was analyzed by ELISA. *p < 0.05; **p < 0.01; ***p < 0.001; ****p < 0.0001. All experiments was analyzed by 2-way ANOVA

We have previously demonstrated that a natural flavonoid, naringenin, can decrease total TGF-β1 secretion through regulation of PKCs [22]. Deservedly, naringenin might reduce the secretion of TGF-β1_EV_ via inhibiting PKC-ζ phosphorylation. Here, our results showed that naringenin had a dose-dependent effect on reducing the level of phosphorylation of PKC-ζ stimulated by irradiation (Fig. 5A) but had no effects on the level of PKC-ζ mRNA expression (Fig. 5B) in 4T1 cells. Furthermore, naringenin markedly attenuated the secretions of the EVs and the TGF-β1_EV_ promoted by radiation (Fig. 5C, D), thus in turn resulting in the prevention of the TGF-β1_EV_-induced Tregs differentiation from CD4^+^ T cells (Fig. 5E). In addition, the in vivo data further demonstrated that naringenin reduced the TGF-β1_EV_ secretion from the irradiated 4T1 tumors (Fig. 5F), indicating that naringenin could be used as an inhibitor of PKC-ζ phosphorylation to combine with radiotherapy as a promising strategy for breast cancer treatment.

### Naringenin reduces PKC-ζ phosphorylation via inhibiting its superoxide-induced release of zinc

**Fig. 6:**
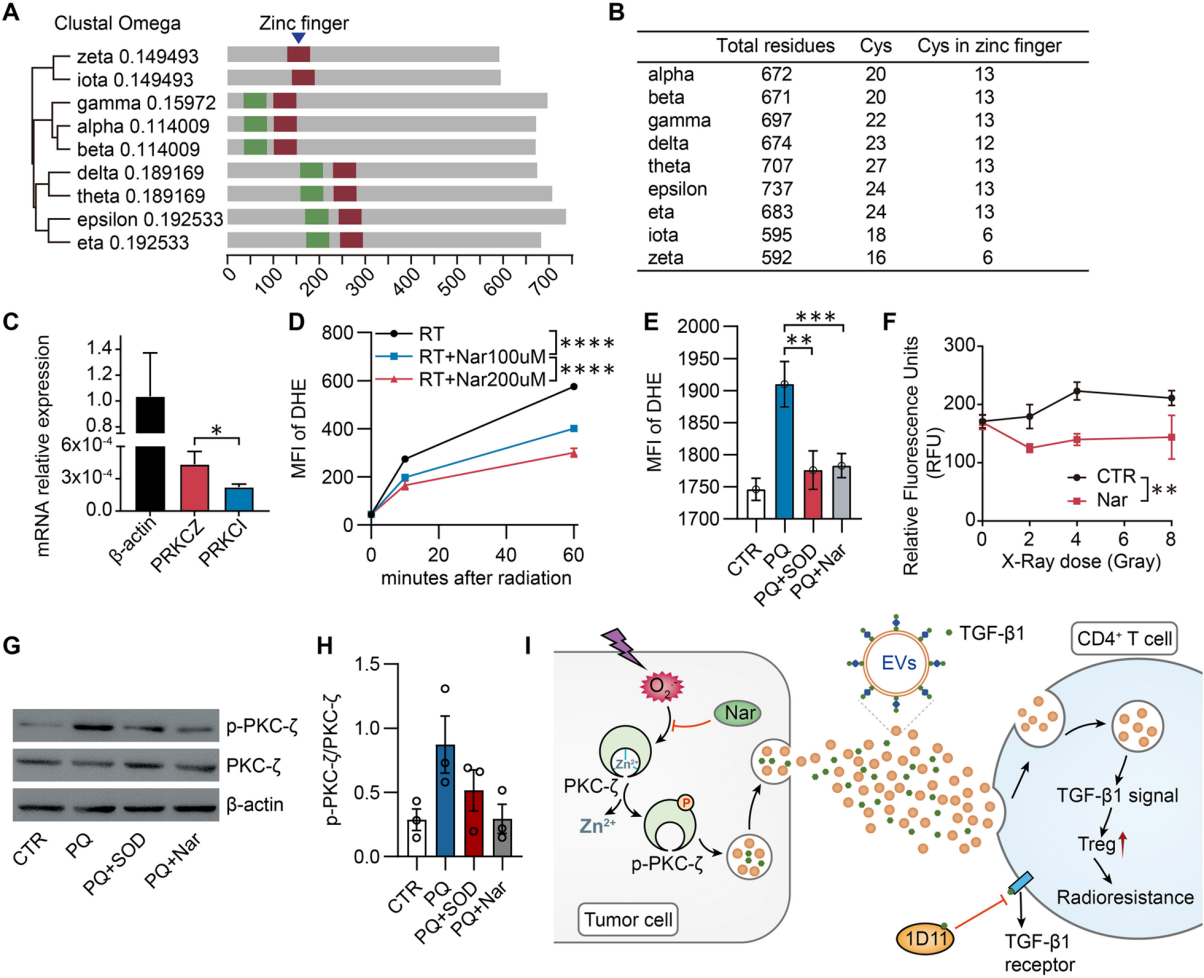
Naringenin mediates the process of superoxide stimulating PKC-ζ activity via the release of Zinc from Zinc finger domain of PKC-ζ. **A** The multiple sequence alignments of different PKC isoforms were carried out using Clustal Omega. The location of the first zinc finger motif is highlighted in green while the second zinc finger motif is highlighted in deep red. The solely zinc finger motif in atypical PKCs is conserved with the second in both classical and novel PKCs, and also highlighted in deep red. **B** Number of cysteines in the whole protein or the zinc finger motif of the cysteine-rich region. **C** PRKCZ and PRKCI mRNA relative expressions in 4T1 cells. Total mRNA was extracted and reverse transcribed into cDNA. Quantitative PCR was used to detect the transcriptional expression levels of PRKCZ and PRKCI genes to β-Actin gene. **D**, **E** Naringenin reduces the elevated superoxide (O2^.^) induced by radiation or paraquat. 4T1 cells were incubated with Dihydroethidium (DHE, 5 µM) for 30 min prior to treatment by radiation (X-ray, 8 Gy). Naringenin (Nar, 100 µM, 200 µM) was added to 4T1 cells for indicated time and MFI of DHC of cells was detected by flow cytometry (**D**). Paraquat (PQ, 100 µM) with or without naringenin (Nar, 100 µM) were added to 4T1 cells for 120 min and MFI of DHC of cells was detected by flow cytometry (**E**). **F** Naringenin inhibits the release of Zinc from 4T1 cells induced by radiation. 4T1 cells were treated with Nar (200 µM) for 30 min. X-Ray of 2, 4 or 8 Gy dose was used to treat 4T1 cells specifically. Cells were stained for 1 min with 150 nM of TSQ dissolved in Lock’s buffer (pH 7.4) and were examined under a fluorescence microscope. **G**, **H** Western blot of total protein lysate from cells treated with paraquat (PQ, 100 μM) and superoxide dismutase (SOD, 500 U/mL) or naringenin (Nar, 200 µM). **I** Model of Naringenin on TGF-β1_EV_ release. Naringenin decreases TGF-β1_EV_ release via the superoxide-Zinc-PKC-ζ-TGF-β1_EV_ release pathway. Following ① radiation inducing superoxide elevation, ② Zinc is released Zinc from the finger domain of PKC-ζ and then PKC-ζ is phosphorylated, ③ which increases the number of releasable vesicles associated with TGF-β1, ④ promotes CD4^+^ T cells uptake, ⑤ activates TGF-β1 signaling, and ⑥ induces Treg cells infiltration and potential radiotherapy resistance. **p < 0.01; ***p < 0.001; ****p < 0.0001. Data were analyzed by *t*-test

To further investigate the regulatory mechanism of naringenin on the TGF-β1_EV_ release when it combined with RT, we need to answer what caused the elevation of PKC-ζ phosphorylation after radiation. It has been reported that PKC can be activated by the exposure to oxidants [29]. In rat brain, the phosphorylated activation of PKC has been thought via oxidation of thiols and release of zinc from the cysteine-rich region [30]. We then assumed that radiation could promote superoxide production, which stimulated PKC-ζ activity via the release of zinc from zinc finger domain of PKC-ζ in breast cancer cells. When the sequences of PKCs were aligned, it showed that, unlike other PKCs, the atypical PKCs (ζ and ι) have only one single zinc finger motif of the cysteine-rich region in the conserved C1 domain (Fig. 6A). The atypical PKCs also has the smallest number of cysteines in zinc finger region among all PKC isoforms (Fig. 6B). However, the two zinc finger motifs in either classical or novel PKCs are tightly bound to each other leading the cysteine residues are partially hidden within the cleft of the binding interface, but the individual cysteine-rich region for the atypical PKCs is highly isolated from other residues (Additional file 1: Fig. S4A, B). Considering that the superoxide has a very short half-life to stimulate the autonomous PKC activity [24], we assume that the atypical PKCs including the ζ and ι isoforms are susceptible for superoxide-induced thiol oxidation and following activity increase. The mRNA relative expression of *PRKCZ* was significantly higher than *PRKCI* in breast cancer cells, including 4T1 cells (Fig. 6C) and MDA-MB-231 cells (Additional file 1: Fig. S5A), which indicating PKC-ζ may be activated by superoxide more easily than PKC-ι.

Radiation is reported to promote TGF-β1 activation through reactive oxygen species (ROS), which is mostly composed of superoxide radicals (O2˙−) [31]. Here we demonstrated that irradiation induced a time-dependent increasing level of O2˙- (DHE staining) (Fig. 6D), a dose-dependent increase of zinc release (Fig. 6F), and an elevated level of PKC-ζ phosphorylation (Fig. 4J), suggesting a positive correlation among O2˙−, zinc release and PKC-ζ phosphorylation. When 4T1 cells were treated with paraquat (PQ), one of the superoxide generators, a marked elevation of O2˙− was observed. Similar to superoxide dismutase (SOD), naringenin reduced the level of O2˙− induced by irradiation or PQ to a relatively low level in a dose dependent manner (Fig. 6D, E). By reducing of the elevated level of O2˙−, naringenin inhibited zinc release (Fig. 6F, Additional file 1: Fig. S5B) and reduced PKC-ζ phosphorylation level significantly (Fig. 6G, H), demonstrating that naringenin decreases the TGF-β1_EV_ release via inhibition of the superoxide-Zinc-PKC-ζ-TGF-β1_EV_ pathway (Fig. 6I). As one of the top transcription factors for *PRKCZ* gene expression (PROMO analysis), NF-kB showed a radiation induced nucleus translocation, which was modulated by naringenin (Additional file 1: Fig. S6). The results suggested that RT induced PKC-ζ activation and then feedback stimulated *PRKCZ* mRNA expression via NF-kB nuclear binding.

### PKC-ζ inhibition overcomes the TGF-β1_EV_ mediated radiotherapy resistance

**Fig. 7:**
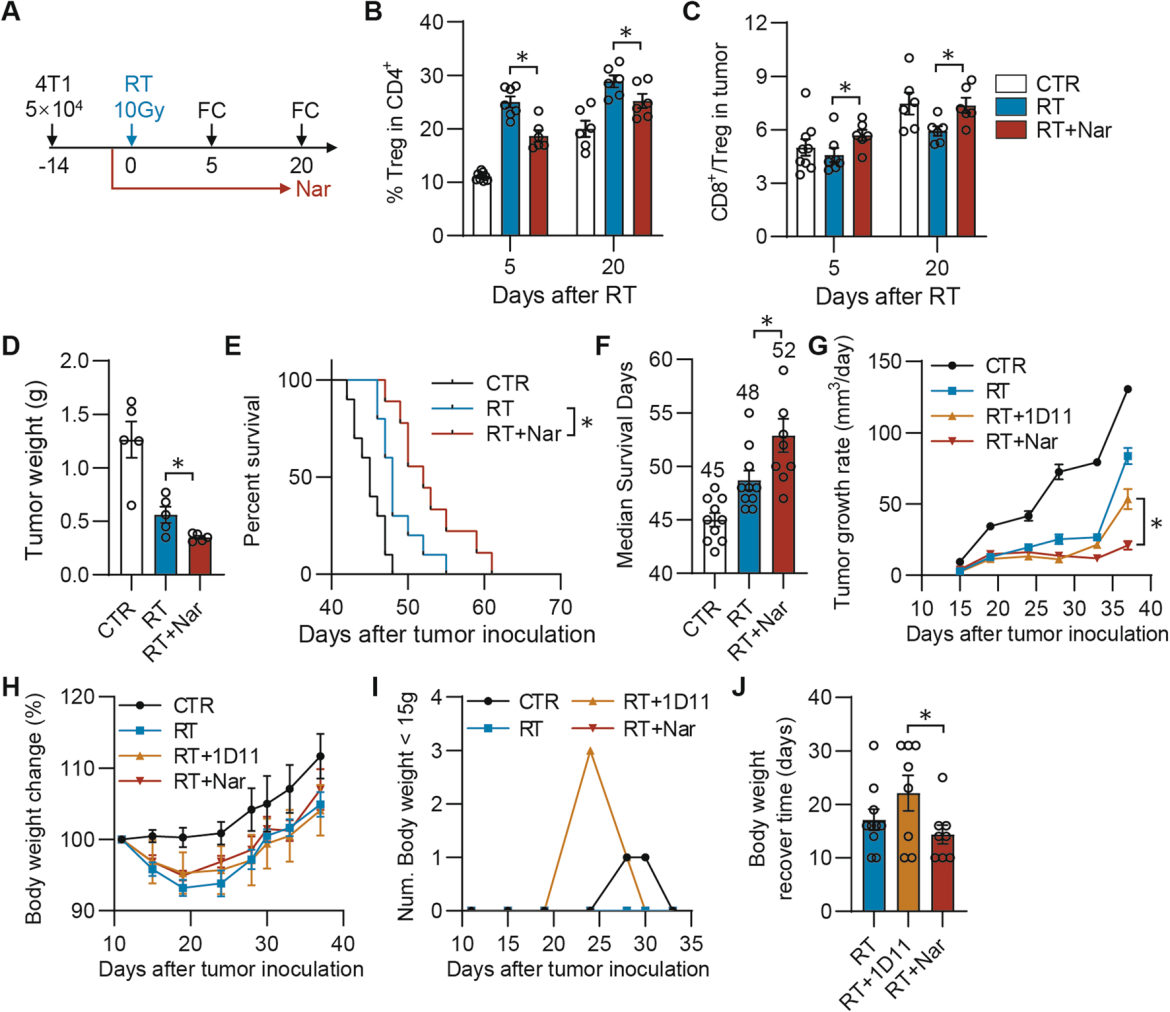
Inhibition of PKC-ζ by naringenin overcomes TGF-β1_EV_ mediated radiotherapy resistance. **A** Schematic diagram of tumor therapy model. Tumor-bearing mice were treated by 10 Gy radiation. Naringenin (Nar, 100 mg/kg, ig) was administrated daily for 30 days. **B**, **C** Tumors were dissected and analyzed by flow cytometry for CD4^+^CD25^+^Foxp3^+^ (**B**) and ratio of CD8^+^/Treg (**C**). **D** The tumor weight on the 35th day after tumor inoculation were measured. **E**, **F** Survival curve and median survival days in groups of mice after different treatment combination. **G** Tumor growth rate in groups of mice after different treatment combination. **H** Rates of body weight changes. Weights of mice calculated as changes from a baseline measurement value before radiotherapy. **I** Number of mice which weight was lower than 15 g after different treatment combination. **J** The days for mice to recover to their body weight before radiation treatment in different groups. *p < 0.05. All experiments was analyzed by 2-way ANOVA

To evaluate the in vivo therapeutic effects of naringenin combined with RT, the 4T1 tumor model was used (Fig. 7A). Compared with RT alone, RT combined with naringenin significantly decreased radiation-induced Tregs infiltration, resulting in a higher ratio of CD8^+^/Treg (Fig. 7B, C). The reduction of suppressive Treg cells in the TME by naringenin could bring an inhibition of tumor weights (Fig. 7D) and a prolonged survival of mice bearing breast tumors (Fig. 7E, F). When compared to 1D11 antibody, naringenin combined with RT was more effective in delaying the tumor growth (Fig. 7G). After RT, the body weights of mice decreased dramatically. The combination of naringenin could restore somewhat of mice weight on the 19 day, 24 day and 37 day after tumor inoculation (Fig. 7H). Specifically, the weights of three mice among ten were lower than 15 g after the mice received RT combining with 1D11 treatments. In contrast, naringenin quickly recovered the mice weight loss caused by radiation and no mouse weight was lower than 15 g (Fig. 7I). A much shorter time was needed to recover for the mice treated by RT combined with naringenin than that with 1D11 (Fig. 7J). Therefore, the inhibition of PKC-ζ to prevent the TGF-β1_EV_ secretion could be an optional combination strategy to overcome the radiotherapy resistance.

## Discussion

Although RT is widely applied to breast cancer patients, radiotherapy resistance is inevitable, presented as the tumor recurrence and poor prognosis. Here we demonstrated that the irradiation preferred to induce an elevated expression and phosphorylation level of PKC-ζ within the TME, resulting in the resistance to radiotherapy by promoting the TGF-β1_EV_ secretion. An effective way to intervene the TGF-β1_EV_ release from breast cancer cells was first put forward in this study.

In clinic, an elevated TGF-β1 level induced by irradiation has been frequently found accumulation in tumor tissues but not in the circulating system [8], suggesting the TGF-β1 is mainly associated with EVs. Not only in breast cancers of mice, but also in human NSCLC tissues, large proportion of TGF-β1 was found associated with the EVs. The fact indicates that TGF-β1 in other types of human cancers may exist in a similar form, which makes the TGF-β1_EV_ widely adapted for other cancers as a promising biomarker. It shows that the high level of intra-tumoral TGF-β1 is usually accompanied with the poor prognosis. Therefore, the TGF-β1_EV_ can also be used as a tumor tissue biomarker to personalize radiation therapy for breast cancers.

Different inhibitors targeting the TGF-β1 pathway have been developed to synergize with radiotherapy in clinical trials, however, the combinational treatments are sometimes with limited efficacies and even less effective outcomes [32]. Here we demonstrated that RT treatment effectively controlled tumor growth but promoted more TGF-β1_EV_ release. Different to the free form TGF-β1, the TGF-β1_EV_ can be endocytosed by receipt cells and transmit intracellular signaling effectively. The failure of the TGF-β1 antibody for the TGF-β1_EV_ blockage possibly resulted in the low efficiency to overcome the TGF-β1_EV_ mediated immunosuppression. Therefore, selective inhibition of the TGF-β1_EV_ release is a promising combination therapeutic strategy for preventing breast cancer progression.

PKC family members are reported to be activated early upon irradiation [33]. Our data provide the further evidence on the importance of superoxide activating PKC-ζ for the TGF-β1_EV_ release, and how naringenin intervene in this process. Although further studies are still needed to determine how naringenin regulates oxidation, our data suggest that naringenin may transform the O2˙- induced by radiation to a non-toxic form, such as H_2_O_2_ [34], and maintain the killing ability of oxidation to tumors. In the study, we demonstrated that irradiation induced a significant increase of PKC-ζ expressions on three levels, including the level of transcription, translation and phosphorylation. Naringenin, however, could block radiotherapy-induced PKC-ζ only on the phosphorylation level to enhance tumor control (Figs. 5A–D and 7). The data indicated that the phosphorylation level of PKC-ζ was particularly important in controlling the release of TGF-β1_EV_.

Preciously, we have demonstrated that naringenin inhibits TGF-β1/Smad3 signaling pathway via the decrease of smad3 expression and can directly block receptor interaction [35, 36], leading to the reduction of Tregs production. Naringenin (YPS345) has been proved to effectively relieve radiation induced pulmonary inflammation and fibrosis [37], which is currently in an ongoing phase II clinical trial in China (NO. CTR20212450). Based on the evidence that naringenin could relieve RT induced toxicity and improve the effectiveness, naringenin is expected to elevate the radiation dose necessary for killing tumors, meanwhile minimizing the side effects of irradiation and overcoming the radiation therapy resistance via the TGF-β1_EV_ intervention. In summary, our data substantiate naringenin to be a promising candidate for the development of potential anti-TGF-β1 agents to overcome radiotherapy resistance.

## Supplementary Information

**Additional file 1:** **Figure S1** EVs promote the differentiation of naïve CD4 + T cells to Tregs. Naïve CD4^+^T cells were isolated from Foxp3-GFP mice and incubated with anti-CD3/CD28 functional antibody with EVs (500 µg/mL) or TGF-β1 (5 ng/mL) for 72 h followed by flow cytometry analysis. **A** The differentiation of naïve CD4^+^ T cells to Treg induced by EVs was detected by quantification of the percentage of CD25^+^GFP^+^cells in CD4^+^ cells. **B** The percentage of Ki67^+^ cells in Treg cells was quantified by flow cytometry analysis. **C** The percentage of Annexin V^+^ cells in Treg cells was quantified by flow cytometry analysis. ****p < 0.0001. All experiments was analyzed by *t* test. **Figure S2** TGF-β1 is largely associated with EVs in 4T1 cells and tumors after radiation treatment. **A** 4T1 cells were treated with 8 Gy radiation and cultured for 48 h. EVs were isolated from supernatant and ELISA was performed for detection of TGF-β1. **B** 4T1 breast cancer tumors carried by C57BL/6 mice were treated with 10 Gy of radiation. Tumors were isolated 14 days after radiation, the tumor tissues were digested and EVs were extracted. Total TGF-β1 and TGF-β1 associated with EVs in the suspension were detected using TGF-β1 ELISA kit. ****p < 0.0001. All experiments was analyzed by 2-way ANOVA. **Figure S3** PKC-ζ siRNA effectively inhibits mRNA and protein expression in 4T1 cells. **A** 4T1 cells were transfected with PKC-ζ siRNA for 24 or 48 h and RNA was isolated for qPCR. **B** Western blot was performed for detection of PKC-ζ protein expression after PKC-ζ siRNA treatment. **Figure S4** 3D structures of PKC isoforms. **A** Structure of the zinc finger motifs of different mouse PKC isoforms adapted from the PKC structure predicted by AlphaFold. The cysteine residues are highlighted in blue. **B** The PKC structure of different mouse PKC isoforms, with the first zinc finger motif highlighted in cyan while the second highlighted in red. **Figure S5** PKCs mRNA relative expression and zinc release in MDA-MB-231 cells. **A** MDA-MB-231 cells were collected and the mRNA relative expression of PRKCZ and PRKCI to β-Actin was analyzed using qPCR. **B** Naringenin (Nar, 200 µM) were added to MDA-MB-231 cells for 30 min before different dose of X-Ray (0, 2, 4 and 8 Gray) administration. The relative fluorescence units were measured by the fluorescence microplate reader. *p < 0.05; **p < 0.01. Data were analyzed by *t*-test. **Figure S6** RT induced the entry of NFkB into nuclear, which was modulated by naringenin. 4T1 cells were treated with 8 Gy of X-Ray (RT) and 200uM of Naringenin (RT + Nar) for 2 h. Total proteins were extracted and isolated into cytoplasmic and nuclear sections. Western blot was performed for detection of NFkB protein expression in nuclear section with β-Actin as its internal reference after different treatment.

## Data Availability

All data associated with this study and/or analyzed during the current study are available from the corresponding author.

## Abbreviations

TGF-β1: Transforming growth factor-β1
EV: Extracellular vesicles
TME: Tumor microenvironment
PKC: Protein kinase C
PKC-ζ: Protein kinase C zeta
Tregs: Regulatory T cells
TGF-β1_EV_: Extracellular vesicle associated-TGF-β1
RT: Radiotherapy
KO: Knockout
WT: Wild type
TCGA: The cancer genome atlas
NSCLC: Non-small cell lung cancer
ELISA: Enzyme linked immunosorbent assay
PMA: Phorbol myristate acetate
CAL: Calphostin C
ROS: Reactive oxygen species
O2˙-: Superoxide radicals
PQ: Paraquat
DHE: Dihydroethidium
SOD: Superoxide dismutase
