# Supplementary material for: PKC-ζ mediated reduction of the extracellular vesicles-associated TGF-β1 overcomes radiotherapy resistance in breast cancer
Source: Breast Cancer Res. 2023 Apr 7;25:38. doi: 10.1186/s13058-023-01641-4 (PMC10082517; doi:10.1186/s13058-023-01641-4)
Supplement: Supplementary file 1 — Additional file 1: Figure S1 EVs promote the differentiation of naïve CD4 + T cells to Tregs. Naïve CD4+T cells were isolated from Foxp3-GFP mice and incubated with anti-CD3/CD28 functional antibody with EVs (500 µg/mL) or TGF-β1 (5 ng/mL) for 72 h followed by flow cytometry analysis. A The differentiation of naïve CD4+ T cells to Treg induced by EVs was detected by quantification of the percentage of CD25+GFP+cells in CD4+ cells. B The percentage of Ki67+ cells in Treg cells was quantified by flow cytometry analysis. C The percentage of Annexin V+ cells in Treg cells was quantified by flow cytometry analysis. ****p < 0.0001. All experiments was analyzed by t test. Figure S2 TGF-β1 is largely associated with EVs in 4T1 cells and tumors after radiation treatment. A 4T1 cells were treated with 8 Gy radiation and cultured for 48 h. EVs were isolated from supernatant and ELISA was performed for detection of TGF-β1. B 4T1 breast cancer tumors carried by C57BL/6 mice were treated with 10 Gy of radiation. Tumors were isolated 14 days after radiation, the tumor tissues were digested and EVs were extracted. Total TGF-β1 and TGF-β1 associated with EVs in the suspension were detected using TGF-β1 ELISA kit. ****p < 0.0001. All experiments was analyzed by 2-way ANOVA. Figure S3 PKC-ζ siRNA effectively inhibits mRNA and protein expression in 4T1 cells. A 4T1 cells were transfected with PKC-ζ siRNA for 24 or 48 h and RNA was isolated for qPCR. B Western blot was performed for detection of PKC-ζ protein expression after PKC-ζ siRNA treatment. Figure S4 3D structures of PKC isoforms. A Structure of the zinc finger motifs of different mouse PKC isoforms adapted from the PKC structure predicted by AlphaFold. The cysteine residues are highlighted in blue. B The PKC structure of different mouse PKC isoforms, with the first zinc finger motif highlighted in cyan while the second highlighted in red. Figure S5 PKCs mRNA relative expression and zinc release in MDA-MB-231 cells. A MDA-MB-231 [file 13058_2023_1641_MOESM1_ESM.docx]

Supplementary Materials for **PKC-ζ mediated reduction of the extracellular vesicles-associated TGF-β1 overcomes radiotherapy resistance in breast cancer**

**Authors:**

Fayun Zhang^1*#^, Zifeng Zheng^1,2#^, Luoyang Wang^3#^, Wenfeng Zeng^1^, Wenjing Wei^1,2^, Chunling Zhang^1^, Ziran Zhao^4*^, Wei Liang^1*^

^#^Co-first author: Fayun Zhang, Zifeng Zheng, Luoyang Wang

* Corresponding author: Fayun Zhang, [fyzhang@ibp.ac.cn](mailto:fyzhang@ibp.ac.cn); Ziran Zhao, [zhaoziran@cicams.ac.cn](mailto:zhaoziran@cicams.ac.cn); Wei Liang, [weixx@ibp.ac.cn](mailto:weixx@ibp.ac.cn).

**The .doc file includes**:

Supplemental Figure 1 to Supplemental Figure 6 and figure legends.

**SUPPLEMENTARY FIGURES**

**
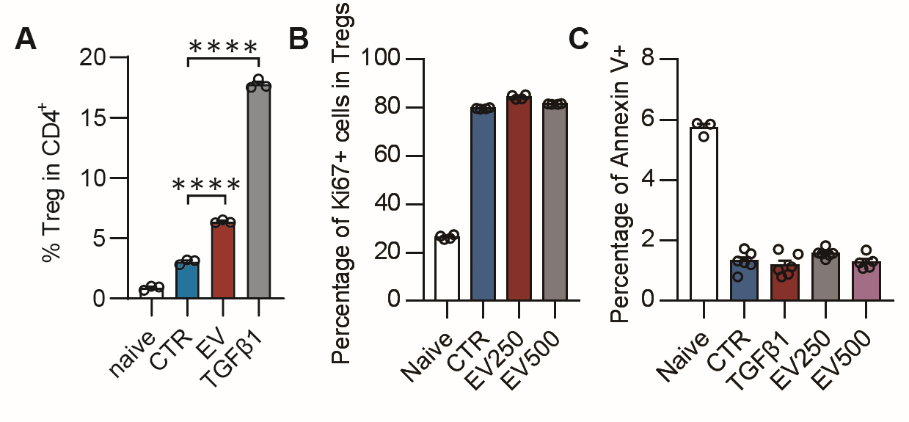
**

**Supplemental Figure 1. EVs promote the differentiation of naïve CD4+ T cells to Tregs.** Naïve CD4^+^T cells were isolated from Foxp3-GFP mice and incubated with anti-CD3/CD28 functional antibody with EVs (500ug/mL) or TGF-β1 (5ng/mL) for 72h followed by flow cytometry analysis. (**A**) The differentiation of naïve CD4^+^ T cells to Treg induced by EVs was detected by quantification of the percentage of CD25^+^GFP^+^cells in CD4^+^ cells. (**B**) The percentage of Ki67^+^ cells in Treg cells was quantified by flow cytometry analysis. (**C**) The percentage of Annexin V^+^ cells in Treg cells was quantified by flow cytometry analysis. ****p < 0.0001. All experiments was analyzed by t test.


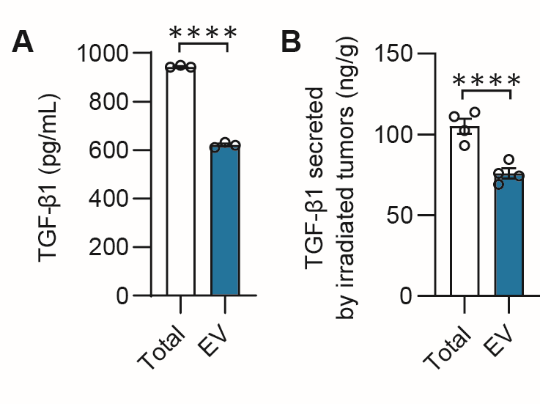


**Supplemental Figure 2.** TGF-β1 is largely associated with EVs in 4T1 cells and tumors after radiation treatment. (**A**) 4T1 cells were treated with 8Gy radiation and cultured for 48 hours. EVs were isolated from supernatant and ELISA was performed for detection of TGF-β1. (**B**) 4T1 breast cancer tumors carried by C57BL/6 mice were treated with 10Gy of radiation. Tumors were isolated 14 days after radiation, the tumor tissues were digested and EVs were extracted. Total TGF-β1 and TGF-β1 associated with EVs in the suspension were detected using TGF-β1 ELISA kit. ****p < 0.0001. All experiments was analyzed by 2-way ANOVA.


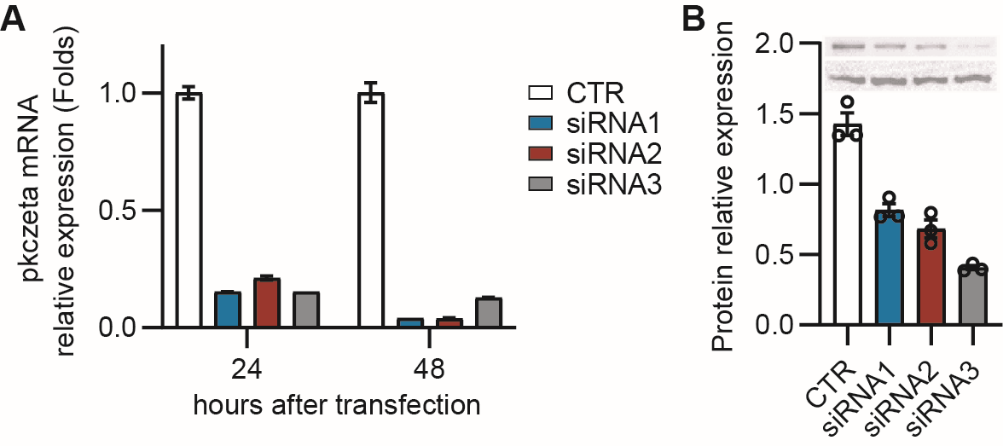


**Supplemental Figure 3. PKC-ζ siRNA effectively inhibits mRNA and protein expression in 4T1 cells.** (**A**) 4T1 cells were transfected with PKC-ζ siRNA for 24 or 48 hours and RNA was isolated for qPCR. (**B**) Western blot was performed for detection of PKC-ζ protein expression after PKC-ζ siRNA treatment.


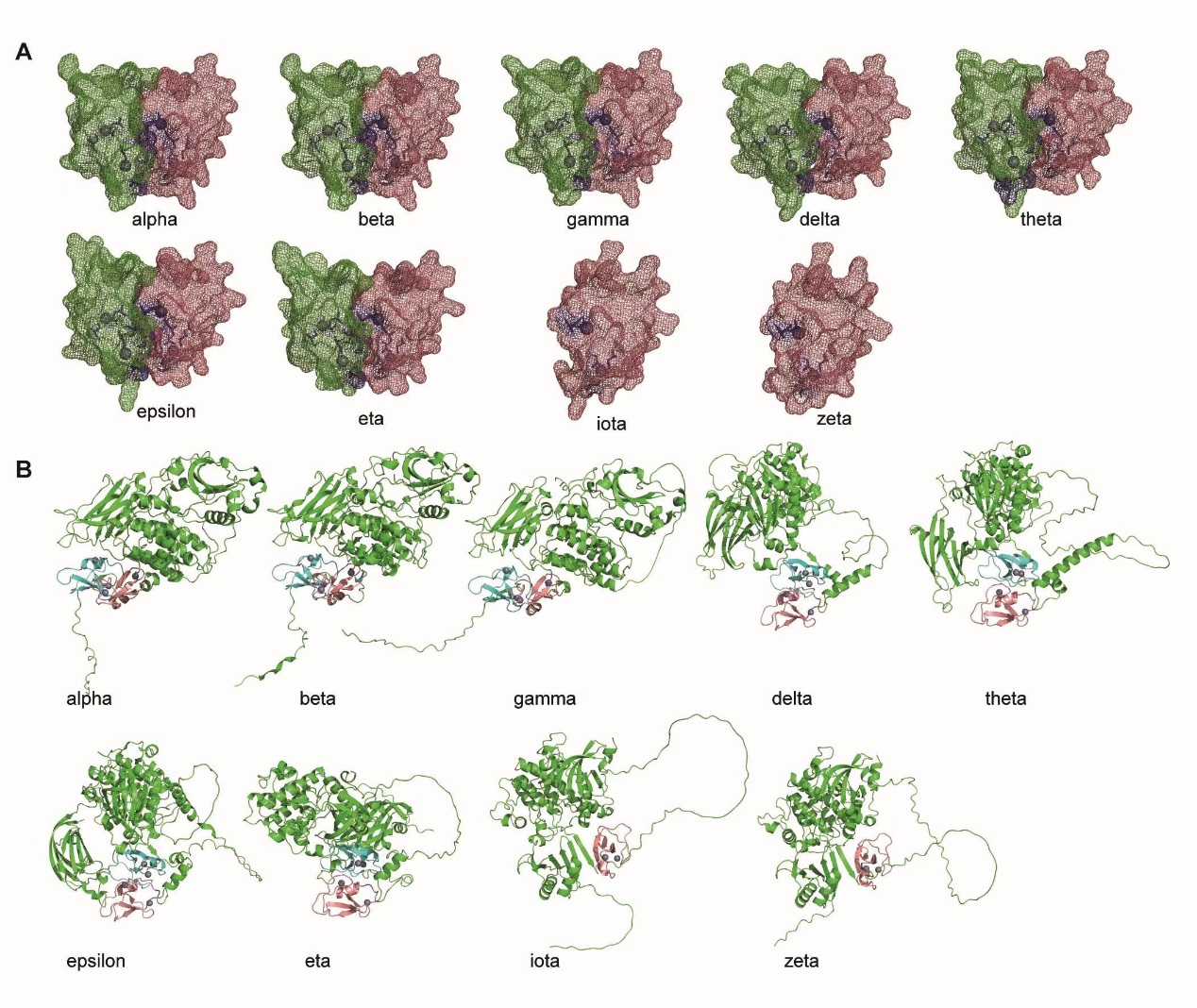


**Supplemental Figure 4.** **3D structures of PKC isoforms.** (**A**) Structure of the zinc finger motifs of different mouse PKC isoforms adapted from the PKC structure predicted by AlphaFold. The cysteine residues are highlighted in blue. (**B**) The PKC structure of different mouse PKC isoforms, with the first zinc finger motif highlighted in cyan while the second highlighted in red.


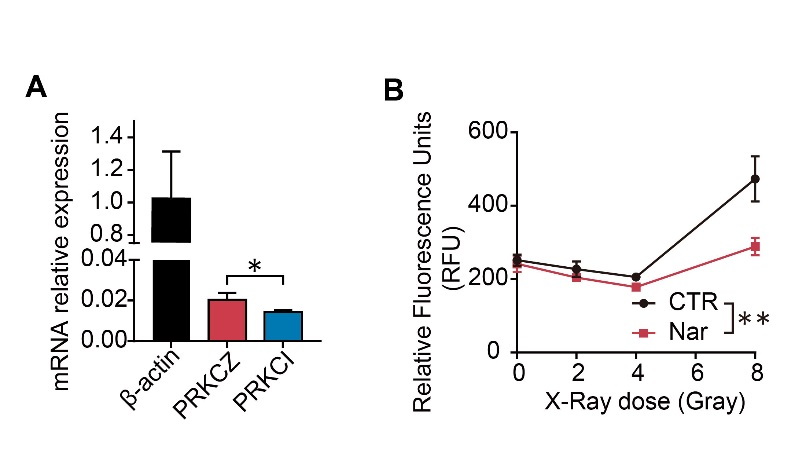


**Supplemental Figure 5. PKCs mRNA relative expression and zinc release in MDA-MB-231 cells. (A)** MDA-MB-231 cells were collected and the mRNA relative expression of PRKCZ and PRKCI to β-Actin was analyzed using qPCR. (**B**) Naringenin (**Nar,** 200uM) were added to MDA-MB-231 cells for 30 min before different dose of X-Ray (0, 2, 4 and 8 Gray) administration. The relative fluorescence units were measured by the ﬂuorescence microplate reader. * p < 0.05; **p < 0.01. Data were analyzed by t-test.

**Supplemental Figure 6. RT induced the entry of NFkB into nuclear, which was modulated by naringenin.** 4T1 cells were treated with 8 Gy of X-Ray (RT) and 200uM of Naringenin (RT+Nar) for 2 hours. Total proteins were extracted and isolated into cytoplasmic and nuclear sections. Western blot was performed for detection of NFkB protein expression in nuclear section with β-Actin as its internal reference after different treatment.
